# Factors Affecting Gynecologic and Sexual Assessment in Older Women: A Lesson for Primary Care Providers

**DOI:** 10.3390/healthcare3030683

**Published:** 2015-08-11

**Authors:** Ayasha Thomason, Natalie Capps, Leanne Lefler, Gloria Richard-Davis

**Affiliations:** 1 College of Nursing, University of Arkansas for Medical Sciences, 4301 West Markham, Slot 529, Little Rock, AR 72205, USA; E-Mails: njcapps@uams.edu (N.C.); leflerleanne@uams.edu (L.L.); 2 College of Medicine, University of Arkansas for Medical Sciences, 4301 West Markham, Slot 518, Little Rock, AR 72205, USA; E-Mail: garicharddavis@uams.edu

**Keywords:** older women, gynecologic screening, guidelines, primary care

## Abstract

Guidelines for screening of cervical cancer and pelvic exams for older women have recently changed. These changes may have unexpected sequelae in women over 65 years of age. This manuscript provides a review of gynecologic screening recommendations for older women in the U.S. and potential ramifications of these recent changes. Peer reviewed guidelines from the American College of Obstetrics and Gynecology, U.S. Preventative Task Force Services, the American Cancer Society, The Centers for Disease Control, and multiple original research articles and reviews were reviewed for this manuscript. Women over 65 are at greatest risk to develop late stage diagnoses of cancers, pelvic organ disease, incontinence, and infections. Clinicians will need to acutely consider this fact when communicating and screening this population. We conclude that practitioners should be aware of the new guidelines and should consider including gynecologic health history and symptom analysis as part of annual exams in women of all ages.

## 1. Introduction

Over the past several years there have been policy changes and updates to practice guidelines in the United States (U.S.) for women’s, particularly older women’s health screenings, namely cervical cancer screening, otherwise known as the Papanicolaou smear (Pap smear) [1]. Often identified as the same exam, the Pap smear and pelvic exam are related, but separate exams. In the Pap smear a small sample of cervical and endocervical cells are taken and examined microscopically for abnormalities such as sexually transmitted diseases, cancers or precancerous cells. Current recommendations in women 30 to 65 include either co-testing with Human Papillomavirus (HPV) test and Pap smear, or HPV only testing as a primary screening option with screening intervals ranging from three to five years [1].

In the pelvic exam, the provider examines the pelvis and its inner structures manually to identify any gross anatomical abnormalities or discomfort [2]. This exam traditionally includes inspection of the external genitalia, bimanual examination of the uterus and adnexa and often a retrovaginal examination. Vulvovaginal inflammation, genital prolapse, postmenopausal bleeding, infection, bladder function alterations and/or gynecologic concerns may be identified incidentally during inspection and bimanual examination. In a cross-sectional analysis of 27,342 women ages 50–79 enrolled in the Women’s Health Initiative, a large population-based study, disorders such as uterine prolapse occurred in approximately 14.2% of women, cystocele in 34.3%, and rectocele in 18.6% respectively [3]. Additionally, about 20 million women in the U.S. have some form of urinary incontinence [4], and approximately 50% of nursing home and assisted living residents are incontinent [5], demonstrating the importance of genitourinary exams and follow-up in older women [6,7,8]. It should also be noted that these rates are in those who voiced symptomatic complaints and therefore represents a bias toward underrepresentation. Nevertheless, prevalence rates of gynecologic issues in older women are significant, and necessitate attention in the form of improving gynecologic assessment and screening methods.

The most important aspect is likely taking an accurate history, performing a symptom analysis if symptoms are reported and using diagnostic reasoning to determine what, if any physical exam techniques need to be performed [9]. However, in older women, recent changes in guidelines may adversely affect the identification and treatment of gynecological disorders. This manuscript provides a review of gynecologic screening recommendations in women over 65 and describes older women’s common gynecologic problems, and discusses possible ramifications if routine screening is eliminated from this population group.

In 2012, the American Congress of Obstetrics and Gynecology (ACOG) and the US Preventative Task Force (USPTF) recommended that women may discontinue having Pap smears at age 65 if: (1) they have negative history of moderate to severe cervical dysplasia in the past 10 years, or (2) after a benign hysterectomy with no history of abnormal pap smears in the past 10 years [10]. Being negative in the past 10 years consists of either three consecutive negative cytology results, or two consecutive negative co-tests [11,12]. The majority of obstetricians, gynecologists, and women’s health nurse practitioners utilize the standards of these two groups and base practice on their guidelines and policy statements. Epidemiologic evidence supports the cessation of annual Pap smears as recommended by these bodies as a means to reduce discomfort, unnecessary overtreatment, reduce costs, and anxiety due to false positive results in older women. However, only limited data provides support for the cessation of pelvic exams [13,14], and there is concern that eliminating pap smears based on data may affect the incidence of pelvic exams. No evidence supports the cessation of a routine gynecologic health history and symptom analysis, and although the guidelines do not support cessation of this examination, it may be incidentally reduced as an outcome of less patient interaction based on new guidelines. Rositch and colleagues (2014) identified the knowledge gap in screening for older women, stating that the “question is not one of overall screening effectiveness, but rather, what age is there a sufficiently low risk in older women with previous normal screening to safely exit them from the screening program” [11] (p. 1). This is important considering that older women are living considerably longer, and the Baby Boomer population is now turning 65 contributing to a large proportion of older women living beyond the recommended discontinuation age [15]. Many health care providers, along with lay people, are confused about the new guideline changes [16,17,18]. Unfortunately, many disease processes do not present with symptoms until in advanced stages [19,20].

The new gynecologic guidelines may lead primary health care providers to see fewer women over age 65 routinely; and as a result, fewer women will have yearly screening mechanisms, as many women use their OB/GYN provider as a primary care provider [17]. Another possible outcome would be eliminating interaction with specialized professionals in gynecology and, as a result, poorer, less complete sexual and gynecological screening. A part of the traditional pelvic exam screens for vulvar, vaginal, and/or ovarian conditions that can be benign, infectious, or malignant and that often have symptomatic complaints. Genitourinary disorders significantly affect the quality of life of older women and may result in reduced function, social activities, and self-esteem [21]. If routine screening for potential problems does not occur, these conditions may be missed by the primary care provider (PCP) and reduce opportunity for early intervention. Further, many older women mistakenly deduce symptomatic complaints to normal processes of aging and often, due to generational and cultural norms, may not openly ask a PCP about urinary or sexual complaints [22,23,24].

## 2. Common Gynecologic Disorders in Older Women

### 2.1. Benign Disorders

Atrophic vaginitis is often a common complaint among older postmenopausal women. Women who are not using hormone replacement therapy or vaginal estrogen have thinning vulvar and vaginal tissues leading to dysparunia, sexual dysfunction, irritation, pelvic floor dysfunction and infection [25]. These complaints may be considered private and older women may be less likely to discuss them [22,23,26]. Nevertheless, if health care providers prompt this population to discuss gynecologic and sexual issues, they may be more likely to mention symptoms and complaints. If women are not seen routinely, or PCPs do not appropriately screen for such issues, they may be reluctant to make an appointment specifically to discuss these types of complaints [27].

Lichen sclerosis (LS) is a common autoimmune “inflammatory dermatosis” of the vulvar skin that may occur in males as well as females. LS usually affects either prepubescent girls or postmenopausal women. The prominent feature is white plaques that can become hypertrophic and extremely itchy, especially at night. Untreated, it can result in fissures and narrowing of the vaginal introitus causing dysparunia. The process causes a scarring like syndrome. To confirm the diagnosis, a biopsy should be obtained, since LS is a precursor for malignant changes. The treatment for this disorder is potent topical corticosteroids. These patients must be monitored closely due to the link between LS and potential vulvar malignancy [25].

Pelvic exams are also important for the identification of pelvic floor concerns such as cystocele, rectocele, and pelvic organ prolapse. These conditions may be socially debilitating due to urinary and fecal incontinence, as well as sexual dysfunction. Pelvic organ prolapse is a common condition in older women, especially post-menopausal women. The absence of hormones can cause significant tissue atrophy that supports many of the pelvic organs [3]. It has been suggested that 45%–76% of patients who seek routine gynecologic care complain of some form of prolapse or incontinence [28]. The changing recommendations may not affect this diagnosis, as it is a common complaint. However, as noted earlier, some women are reluctant to openly discuss pelvic floor issues with a PCP unless they are specifically asked about them [29]. Women may go for long periods of time without treatment for these conditions, or seek treatment only after a condition becomes unbearable and early treatments are missed. In fact, women on average wait 6.5 years from the time they first have symptoms to get a diagnosis for bladder issues, and two-thirds of those who leak urine do not use any treatment [30].

Pelvic organ prolapse has long been a problem referred for surgical correction. However, there are less invasive measures that can be utilized by the PCP as initial management before referring for surgery. One such method is the use of pessaries. The pessary is an older method of protecting the weak vaginal wall during most daily activities to improve pain, pressure, sexual issues, and incontinence problems. However, pessaries can be used only for mild to moderate forms of pelvic organ prolapse. Another method of improving prolapse and incontinence issues is physical therapy, which employs behavioral or physical exercises with the use of biofeedback [28]. However older women in general have little knowledge of these treatments unless regular communication occurs with a PCP.

### 2.2. Infectious Disorders

Older women are at increased risk for sexually transmitted infections (STI) and bacterial infections, if exposed, due to natural physiologic aging and thinning of the vaginal mucosa. Due to estrogenic decline and loss of the epithelial cell cascade that produces the healthy protective bacteria, Lactobacillus, older women tend to have thinner vaginal tissues and increased vaginal pH that may result in bacterial overgrowth [22]. In addition, these thinner tissues may be prone to trauma whereby bacteria or viruses could invade and cause infection. Older women who do not protect themselves during new sexual encounters are placing themselves at high risk of transmission of STIs. It has been suggested that older women do not feel a need for protection since they can no longer conceive [31,32]. Risky sexual behavior in older adult women has recently been identified as more common than once thought. Olivi, Santana, and Mathias (2008) assessed knowledge of the risk of STIs in older adults in 165 educated men and women over the age of 50, and 92 agreed that condoms prevented STIs and AIDS. Thus they had some knowledge of the connection between barrier contraception and the prevention of STIs and AIDS, however; only 13.3% always wore condoms with intercourse [33]. From this small population set, it might be deduced that these patients perceive their risk of acquisition of HIV as low. It is currently recognized that older women, especially minority older women account for the highest rates of those living with HIV in the US. Older adult black women have 12 times higher risk for contracting HIV than their white counterparts [34]. Testing for STI is usually not offered since there is no recommendation for routine testing of STIs in this population [35]. As a result, many STIs are at later stages when diagnosed [36].

Recently, the rates of the STI Trichomoniasis vaginalis have increased among the older population. Trichomoniasis has a strong estrogen receptor affinity, increasing the risk of transmission in women who are on estrogen replacement therapy [35]. There is no mandate to report Trichomoniasis, therefore, statistical reports are likely to underrepresent incidence. A recent study found that that among 7593 women age 18–89, women 50 and older had the highest Trichomoniasis rate, at 13% [35]. Older women, however, have fewer signs and symptoms of Trichomoniasis or may even be asymptomatic, making diagnosis difficult. Further, the Centers for Disease Control do not have standard recommended testing intervals and Trichomoniasis is a known cofactor for transmission of HIV. Studies have shown that Trichomoniasis increases susceptibility of contracting HIV in an uninfected partner, and increases infectivity of HIV positive persons. It is thought that Trichomoniasis increases the viral shedding of HIV in females, and increases HIV RNA in male semen almost six-fold [37,38]. There is concern that unprotected older adult intercourse, coupled with decreased gynecologic care may cause poor health outcomes in this population.

Human Papillomavirus (HPV) is a powerful player in dysplastic changes in cervical and vulvar cells. There are over 70 HPV types, and at least 10 are high-risk types. High-risk strains have the potential to cause dysplastic cellular changes that can lead to cancer. HPV infection may be transient or persistent. Transient infections are common in younger women with healthy immune systems. This type of infection may cause dysplastic changes that return to normal over a period of time. Persistent HPV infection is less common but more severe in terms of dysplastic cellular changes [39]. Older women with compromised immune systems may have more problems associated with persistent infection. Aging alone causes a general decrease in cellular and humoral immunity, making older women more susceptible to adverse outcomes related to persistent HPV infections [22]. Grainge *et al*., (2005) found in a study of 710 women, that 21.3% of women over age 51 who were HPV negative at baseline, had HPV three years later [40]. The incidence was higher in this group than younger age groups, suggesting that older women are engaging in sexual activities that place them at risk for HPV [40]. A recent large population based study found high risk HPV prevalence to be 6% in women aged 57–85, and that these rates increased with sexual frequency, marital status, smoking status, hysterectomy, and history of cancer [41]. With increasing HPV infections in women over 65 and the potential for reduced routine screening without assessment of new sexual partners, a significant increase in HPV related diseases and related sequel are likely.

### 2.3. Malignant Disorders

According to the American Cancer Society (ACS), age is the single most important risk factor for development of vulvar cancer; and more than half are diagnosed after the age of 70 [42,43,44]. Although HPV has been linked to 50% of vulvar cancers, these cancers are most commonly found among younger women [43]. Non-HPV related forms of vulvar cancers primarily affect older women. Ueda *et al*., (2011) [45] have suggested that there are two pathways for the development of vulvar cancers. The first is HPV mediated, and the second is associated with lichen sclerosis, which is usually associated vulvar cancer in the older adult. Currently, the only vulvar cancer screening method is symptom-based examination including visualization and inspection of the vulva and vagina [25]. If discontinuation of routine pap smears and pelvic exams occur in older women, routine inspection is likely not to occur. Recent guidelines are unclear on how often visual inspection examinations should now occur [9,12,46,47]. With a push toward more HPV focused testing, screening protocols for vulvar cancers should be further reviewed and more research conducted to determine protocols for screening older women for vulvar and vaginal cancers. There is early evidence linking HPV to some types of oral and anal cancers, and this should be examined as an emerging trend [48,49].

Research has found that women who smoke have up to a five-fold increase in risk of cervical cancer [50,51] and presently 19.1% of women in the U.S. smoke [52]. Older women who were previous smokers and second hand smoke exposed are also at increased risk cervical dysplasia and therefore should be screened more carefully than those without other risk factors [53]. In addition, a genetic component identified in some women increases the risk of cervical cancer [54]. This link is likely enhanced by cigarette smoking [53]. Thus, women who smoke should be considered at higher risk for vulvar and vaginal cancers in addition to cervical cancers. These women might be considered a sub-population who should be screened more often and monitored closely.

## 3. Long-Term Care Settings

As older women live well beyond 65 years of age, many are in long term care settings, and must rely on staff in the facilities to perform health assessments. Sexual health in long-term care settings is of great concern since the baby boomer population of older adults is moving to these high-density dwelling spaces. There are approximately one million older adults living in assisted living settings, and 1.4 million in nursing homes, and this number will continue to grow in the coming years [7,55,56]. Identifying potential gynecologic concerns with referral to an appropriate health care provider will likely become the burden of long term care facilities. Women in settings without easy access to gynecologic care such as nursing homes and assisted living centers, are at a particular disadvantage since there is no recommended routine examination or screening for vulvar, vaginal, and ovarian cancer. These women may not have exams that include visual inspection, raising concerns that gynecologic problems and cancers might go undiagnosed in this population group. Additionally, many of the residents suffer from reduced cognition, possibly altering the ability to complain of symptoms. Because the new trend of screening leans towards symptom-based management, these disorders may be profoundly problematic in long-term care settings.

There is a documented need for better assessment skills among staff in nursing homes and assisted living centers regarding sexuality, and physical well-being [57]. The Future of Nursing report by The Institute of Medicine pushed for more advanced degree nurses in all areas of nursing as one strategy to improve health care [58]. However, certified nursing assistants with limited training are often utilized in long-term settings as cost saving strategies. Subsequently, the potential for a delay in identifying vulvo-vaginal issues is likely.

## 4. Health Care Providers and Utilization

General practitioners may avoid sexual health questions and gynecologic examinations in older women [59]. As a result, many older women are not screened or educated about gynecological health adequately. The frequency of sexual healthcare concerns of women aged 65 and older is reported as similar to those of younger women, although the type of concerns may vary [60,61]. Often the older the healthcare provider, the less likely they are to ask about vaginal or vulvar issues and/or sexual dysfunction/function [62]. Because of reduced perceived competency in addressing sexual health concerns, reduced understanding of relevant psychosocial issues associated with sexual health, and a perceived or actual reduction in clinical management skills addressing sexual health, many practitioners avoid routine assessment of sexual health [62]. Older women themselves also tend to dismiss complaints of this nature and attribute them to normal aging processes, or hesitate to raise concerns [22,63]. However, if the health care provider is female, there tends to be more communication regarding gynecologic issues and sexuality [64,65]. Cervical cancer increases with age, lower socioeconomic class, and minority status, indicating disparities in access and utilization of gynecologic and/or associated risk care [66].

## 5. Conclusions

Rapid increases in the number of older aged women presents significant challenges for health care delivery. As discussed in this paper, many factors affect the identification and treatment of gynecological conditions in older women. Decreased gynecologic screening has the potential to become problematic for older women, as we know women over 65 remain at the highest risk for benign, infectious, and malignant disorders that decrease functionality and/or longevity, if left untreated. Eliminating Pap screening for women over 65 is likely to decrease unnecessary examinations yet there is concern that Pap smear elimination will erroneously indicate that women over 65 no longer require gynecological care. Simply performing a gynecological health history and asking age-appropriate questions may be the most important tool for the PCP. This simple, low cost, intervention may help in eliminating any adverse outcomes from newly established guidelines. Establishing rapport is integral to asking about urinary habits, leaking, pain, discomfort, discharge, as well as sexual problems and new sexual partners to allow older women to feel that it is acceptable to talk about gynecological health.

Providing adequate information is imperative for women to understand that even though they are not at risk for pregnancy, precautions must be taken to avoid sexually transmitted diseases. Early identification and timely intervention are effective forms of preventative care and can make the difference in quality of life for many older women suffering from gynecologic complications. Older women residing in nursing home facilities and assisted living settings provide even greater challenges to providers. Instituting a treatment or referral protocol in long term care facilities might significantly reduce the incidence of pelvic floor cancers and other issues. We have discussed how early identification of gynecological symptoms can improve function, reduce social isolation, and may improve quality of life. We caution that age should not be the deciding factor that prevents a routine diagnostic and therapeutic approach to gynecologic care.
